# A qualitative analysis of loss-related memories after cancer loss: a comparison of bereaved people with and without prolonged grief disorder

**DOI:** 10.1080/20008198.2020.1789325

**Published:** 2020-09-23

**Authors:** Kirsten V. Smith, Harriet Rankin, Anke Ehlers

**Affiliations:** a Department of Experimental Psychology, University of Oxford, Oxford, UK; b Oxford Health NHS Foundation Trust, Oxford, UK; c The Loss Foundation, [Registered Charity 1147362], London, UK

**Keywords:** Cognitive behavioural therapy, grief, memories, trauma, bereavement, Terapia cognitivo-conductual, pérdida, recuerdos, trauma, duelo, 认知行为疗法, 哀伤, 记忆, 创伤, 丧亲, • We detail the content, qualities, and triggers of loss-related memories in people bereaved by cancer. • Results suggest that individuals suffering from PGD/PCBD experience more upsetting loss-related memories that are predominantly negative in nature. • Nearly all participants reported experiencing intrusive loss-related imagery suggesting that clinical approaches that include memory processing may be particularly relevant for individuals bereaved by cancer.

## Abstract

**Objective**: The study aimed to explore the content and features of loss-related memories in a sample of individuals bereaved by cancer with and without a probable diagnosis of prolonged grief disorder/persistent complex bereavement disorder (PGD/PCBD).

**Methods**: Semi-structured interviews with 28 bereaved adults (PGD/PCBD = 12, NoPGD/PCBD = 16) were analysed using thematic analysis.

**Results**: Three superordinate themes were identified: (1) intrusive imagery, (2) qualities of memory, and (3) triggers. Results showed that individuals suffering from probable PGD/PCBD reported a predominance of negative and upsetting memories, happy memories triggering pain and more negative intrusive imagery than those without PGD/PCBD.

**Conclusions**: Bereavement by cancer can result in troubling intrusive memories that overshadow positive memories. Sufferers of PGD/PCBD are more likely to experience loss-related memories as negative and upsetting. Clinical approaches that utilise memory processing may be of particular relevance in this group.

## Abstract (es)

**Objetivo**: El estudio tuvo como objetivo explorar el contenido y las características de los recuerdos relacionados con la pérdida en una muestra de personas viviendo un duelo por cáncer con y sin un diagnóstico probable de trastorno de duelo prolongado/trastorno de duelo complejo persistente (PGD/PCBD).

**Métodos**: Se analizaron entrevistas semiestructuradas con 28 adultos dolientes (PGD/PCBD = 12, NoPGD/PCBD = 16) mediante análisis temático.

**Resultados**: Se identificaron tres temas supraordinados: (1) imágenes intrusivas, (2) cualidades de la memoria y (3) gatillantes. Los resultados mostraron que las personas que padecen un probable PGD/PCBD informaron un predominio de recuerdos negativos y molestos, recuerdos felices que desencadenan dolour y más imágenes intrusivas negativas que aquellos sin PGD/PCBD.

**Conclusiones**: El duelo por cáncer puede provocar recuerdos intrusivos problemáticos que eclipsan los recuerdos positivos. Las víctimas de PGD/PCBD tienen más probabilidades de experimentar los recuerdos relacionados con la pérdida como negativos y molestos. Los enfoques clínicos que utilizan el procesamiento de memoria pueden ser de particular relevancia en este grupo.

## Abstract (zh)

**目的**: 本研究旨在探讨在患有或未患有可能诊断为延长哀伤障碍/持续性复杂丧亲障碍 (PGD/PCBD) 的癌症患者中, 丧亲相关记忆的内容和特征。

**方法**: 采用主题分析法, 对28名丧亲的成年人 (PGD/PCBD = 12, 无PGD/PCBD = 16) 进行半结构式访谈。

**结果**: 确定了三个上级主题: (1) 闯入性图像, (2) 记忆质量, 和 (3) 触发因素。结果表明, 与无PGD/PCBD者相比, PGD/PCBD患者可能会表现出负性和沮丧的记忆, 触发痛苦的开心记忆以及更多的负性闯入性图像。

**结论**: 癌症丧亲可能导致恼人的闯入性记忆, 比正性记忆更多。 PGD/PCBD患者更可能经历负性和沮丧的丧亲相关记忆。对于此群体, 利用记忆处理的临床方法可能特别适用。

## 1. Background

The loss of a significant other following cancer presents the bereaved individual with a series of emotional, practical, social, and health stressors. While most of these individuals will adapt well following their loss (Guldin, Vedsted, Zachariae, Olesen, & Jensen, 2012; Thomas, Hudson, Trauer, Remedios, & Clarke, 2014) some will go on to experience negative grief-related mental health symptoms that have been conceptualised as prolonged grief disorder (PGD) in ICD-11 (World Health Organization, 2018) or persistent complex bereavement disorder (PCBD) in DSM-5 (American Psychiatric Association, 2012). Although recent research has suggested that the different diagnostic criteria and classifications vary in terms of prevalence and validity (Lenferink, Boelen, Smid, & Paap, 2019), conceptual models of the development and maintenance of both PGD and PCBD have proposed similar maintenance factors, including an important role of memory processes. It is suggested that symptoms of PGD/PCBD arise from a failure to integrate information about the loss into the existing autobiographical memory base (Boelen, van den Hout, & van den Bout, 2006; Maccallum & Bryant, 2013) or attachment-related long-term memory (Shear et al., 2007). This failure of memory integration is central to several models of PGD/PCBD. Shear and colleagues proposed that grief symptoms arise as a result of a mismatch between the reality of the death and the mental representation of an attachment figure as being both emotionally and proximally available (Shear et al., 2007). Boelen et al. (2006) similarly suggest a failure to integrate the reality of the loss into the person’s existing mental representation of his/her self and the world.

Studies investigating this failure of loss integration have found impairments in autobiographical memory specificity in PGD/PCBD sufferers compared to bereaved controls (Dalgleish, Rolfe, Golden, Dunn, & Barnard, 2008; Golden, Dalgleish, & Mackintosh, 2007; Maccallum & Bryant, 2010b). These impairments are understandable since the autobiographical memory system is thought to develop, express, and maintain the self (Conway & Pleydell-Pearce, 2000), aid in the contextualisation of the past, present, expected future, and assist in the development of goal oriented behaviours (Williams et al., 2007). All of these processes are likely to be drawn upon during healthy adaptation to bereavement.

Another area of memory investigated in bereavement is intrusive images. Boelen and Huntjens (2008) investigated the types of intrusive images experienced in a bereaved sample and found the frequency of negative intrusions (i.e. re-enactment fantasies, unpleasant future images, and unpleasant moments surrounding the death) to be correlated to symptoms of PGD, depression, and anxiety, suggesting intrusions are common in bereavement-related mental health problems.

Despite these advances in our understanding of memory processes in grief, little is known about the characteristics, content, triggers and qualities of loss-related memories, features that may point to specific failures of loss integration. Here we aimed to use semi-structured interviews to explore the features of loss-related memory in a sample of bereaved individuals with and without probable PGD/PCBD who had lost loved ones to cancer. A recent study suggested that previously proposed criteria for PGD (Prigerson et al., 2009) and PCBD (American Psychiatric Association, 2012) are diagnostically substantively the same with strong diagnostic properties (Maciejewski, Maercker, Boelen, & Prigerson, 2016). However, PCBD requires that 12 months have passed since death to qualify for a diagnosis, while PGD stipulates only 6 months are necessary. As our sample were 6 months or longer post loss we primarily used the criteria for PGD proposed by Prigerson et al. (2009) and cross-validated our findings using the minimum symptom criteria for PCBD (American Psychiatric Association, 2012).

## 2. Methods

### 2.1. Participants

Participants were 28 adults bereaved by cancer at least six months prior to participation. Participants were recruited from bereavement charity mailing lists and social media as well as the Google content network. Fifty-two participants were invited to take part in qualitative interviews about their grief. Thirty-one participants indicated they were interested in being interviewed and twenty-eight were interviewed. Individuals who expressed concerns about their ability to cope with the emotional content of the session in a screening phone call at recruitment were excluded from taking part. One participant was excluded on this basis. All participants completed online questionnaires (Qualtrics, 2005) before the interview session. The mean time from loss to interview was 27.4 months (SD = 18.78, range: 6–90 months, *Mdn *= 20).

### 2.2. Symptom measures

#### 2.2.1. Adapted prolonged grief disorder inventory (PG-13)

The PG-13; Prigerson and Maciejewski (2008) assesses the prevalence and severity of PGD symptoms (e.g. yearning for the deceased, feelings of emotional numbness/detachment from others, feeling that a part of oneself died along with the deceased). A probable diagnosis of PGD was defined as meeting at least one item of separation distress, and at least five out of nine symptoms of cognitive, emotional or behavioural disturbances daily or several times a day for at least six months following the bereavement (Prigerson et al., 2009). These difficulties also had to result in a significant impairment in social, occupational or other important areas of functioning (e.g. domestic responsibilities).

To validate our results against the PCBD criteria six additional items were added to the PG-13 to correspond with the PCBD criteria not represented by the PGD-2009 criteria.^1^ The PCBD criterion was met if participants endorsed at least one item of separation distress daily (criterion b), at least six experiences of cognitive, emotional, and behavioural symptoms at least ‘once a day’ or ‘quite a bit’ (criterion D), and symptoms resulted in significant impairment in functioning.

#### 2.2.2. *Posttraumatic stress disorder checklist for DSM-5 (PCL-5)* (Weathers et al., 2013)

The PCL-5 is a self-report instrument assessing distress associated with the 20 symptoms of PTSD according to DSM-5 over the past month. Items are rated on a five-point scale, from 0 (not at all) to 4 (extremely). A cut-off score of 33 has been recommended for a probable PTSD diagnosis. Participants were asked to complete the PCL with reference to the death of their significant other.

#### 2.2.3. *Patient health questionnaire (PHQ-9)* (Kroencke, Spitzer, & Williams, 2001)

The PHQ-9 is a self-report measure based on the (DSM IV-TR, American Psychiatric Association, 2000) for major depressive disorder. It assesses nine depressive symptoms, with each item scored 0 (not at all) to 3 (nearly every day) in the last two weeks. A cut-off score of 9 has been recommended for a probable diagnosis of depression.

##### 2.2.3.1. Interview schedule

A semi-structured interview schedule^2^ was developed with input from therapists experienced in the treatment of chronic grief reactions and through a review of the literature (Boelen et al., 2006; Shear et al., 2007). It was designed to extract relevant information regarding unintentional and intentional loss-related memories (e.g. ‘Do you have memories of the deceased that pop into your mind repeatedly?’). In line with case formulation in cognitive-behavioural therapy (CBT) participants were prompted for information regarding the content and qualities (‘Can you tell me more about these memories?), antecedents (“What triggers these memories?”), and consequences (What do you do when these memories come to your mind?’). The theoretical background of these questions assumes that participants’ memories, thoughts, behaviours and emotions interact and influence one another (Beck, 1979; Ehlers & Clark, 2000). This paper describes the content of these interviews that pertains to information related to the triggers, qualities, and content of loss-related memories.

### 2.3. General procedure

The research was reviewed and approved by the Oxford University Medical Sciences Central University Research Ethics Committee (Reference MS-IDREC-C1-2015-032). Participants received electronic information sheets and informed consent was obtained from the participant before completing the online measures. Interviews were conducted by the first author and all sessions were audio recorded. After data collection, participants were debriefed and given a research support leaflet detailing relevant free services. Participants were sent a ‘check in’ email the next day to see if there were any aspects of the session or their reaction that they would like to discuss (Smith, Thew, & Graham, 2018).

### 2.4. Data analysis

All sessions were transcribed verbatim and analysed using a thematic analysis approach and the software package NVivo 7 (www.qsrinternational.com). Code and theme development was conducted following the guidance by Braun and Clarke (2006), emphasizing familiarity with the data, and the iterative refinement of themes. Similarities between themes were identified and aggregated to develop superordinate and subordinate themes. For each theme a file of transcript extracts was created. Themes and exert examples were developed and refined by the first and last author. Towards the end of the qualitative analysis process no new categories were created, suggesting that all major themes had been identified. Each transcript was then re-read to determine whether newer themes more clearly captured previously coded sections. Any individually coded items not relevant were removed from the broader themes. An independent rater reviewed 10% of the extracts and their corresponding themes (Barbour, 2001). Inconsistencies were reviewed and areas were reconsidered until consensus was achieved. Demographic information and psychopathology symptoms severity was subject to significance testing by group using t-tests for continuous data, rank order correlations for ordinal data, and chi-square for categorical data.

## 3. Results

### 3.1. Participants

#### 3.1.1. Demographic characteristics

Characteristics of participants with and without probable PGD are reported in Table 1. Of the 12 participants that met criteria for probable PGD (Prigerson et al., 2009), 92% (*N* = 11) also met criteria for probable PCBD (American Psychiatric Association, 2012). The remaining participant met the minimum symptom criteria for PCBD but was only 11 months post-loss. Given that it was likely that they would still meet criteria in a month’s time we included them in the PGD/PCBD analysis. No participants classified as non-clinical using the PGD conceptualisation met criteria for PCBD.

**Table 1.** Participant demographics, loss characteristics, and psychopathology.

	Interview Sample		
	PGD/PCBD (*n* = 12)	NoPGD/PCBD (*n* = 16)	Statistic (*t, r* or χ^2^)
**Demographics**			
Age in years *M (SD)*	59.00 (6.38)	54.50 (11.55)	*t* = -1.21
Gender *N (%)*			
Female	10 (83.33)	12 (75.00)	.673^a^
Highest level of education *N (%)*			
No qualifications	1 (8.33)	0 (.00)	*r* = -.42*
High school education	4 (33.3)	2 (12.5)	
Undergraduate degree	6 (50.0)	8 (50.0)	
Postgraduate degree	1 (14.3)	6 (37.5)	
**Loss characteristics**			
Months since loss *M (SD)*	28.33 (15.48)	26.06 (21.37)	*t* = -.31
Who died*? N (%)*			
Spouse/Partner	10 (83.33)	11 (68.75)	
Child	1 (8.33)	1 (6.25)	
Parent	0 (.00)	2 (12.50)	
Other	1 (8.33)	2 (12.50)	
Length of relationship *M (SD)*	332.41 (160.60)	365.00 (133.03)	*t* = .59
in months			
**Psychopathology Symptom Severity**			
PGD (PG-13) *M (SD)*	45.83 (5.24)	30.31 (7.40)	*t* = -6.18***
Depression (PHQ-9) *M (SD)*	18.67 (5.69)	8.43 (5.80)	*t* = -4.70***
PTSD (PCL-5) *M (SD)*	46.58 (15.19)	23.59 (12.69)	*t* = -4.36***

^a^significance of Fisher’s Exact Test (2x2). PG-13 = Prolonged Grief Disorder Scale, PHQ-9 = Patient Health Questionnaire, PCL-5 = Posttraumatic stress disorder Checklist for DSM-5.

*p* <.001***

Participants with and without probable PGD/PCBD did not significantly differ in terms of age, gender, months since death or length of relationship. However, groups differed with respect to education, with the PGD/PCBD group reporting lower levels of education. As to be expected, they differed significantly on scores of grief symptom intensity as assessed by a continuous measure derived from the PG-13 (Prigerson & Maciejewski, 2008), depression (Kroencke et al., 2001), and PTSD severity (Weathers et al., 2013). Two thirds (67%) of the PGD/PCBD group also met criteria for a probable diagnosis of PTSD.

### 3.2. Semi-structured interview – principal themes

The thematic map listing all superordinate and subordinate themes is presented in Table 2.

**Table 2.** Thematic map of superordinate and subordinate themes.

Superordinate theme	Subordinate theme
Intrusive imagery	Change for the worse
	Illness-related imagery
	Positive past memories
	Deceased in the present
Qualities of memories	Negative memories take precedent Distressing Happy memories cause pain ‘Nowness’ Vividness Felt Presence
Triggers	Time markers Couples and families Illness or death-related triggers Internal physical or emotional states Music Possessions and photographs Shared, locations, events, and activities They would have done/thought/been here When distraction comes to an end

#### 3.2.1. Intrusive imagery

The majority of the sample (89.3%) reported current intrusive memories relating to circumstances surrounding the death as well as the death event when asked about memories that pop into their mind repeatedly.

##### 3.2.1.1. Changed for the worse/loss of hope

Two thirds of the PGD/PCBD group (66.7%) and 43.8% of the NoPGD/PCBD group reported intrusive memories relating to traumatic time points common in cancer treatment (first diagnosis, terminal diagnosis, moment of death); these moments appeared to signal a loss of hope or the moment the prognosis changed for the worse.

First diagnosis:

‘The very moment that they said that he had a brain tumour and his reaction. He was just so quiet and dignified. Because when you see a consultant running down the corridor trying to find you before you leave an MRI unit; you know it’s not going to be good news.’ [P1]

Terminal diagnosis:

‘It’s the moment that I thought he still looked well and they told me he had less than a month to live. And he didn’t know; they hadn’t told him; and I had to explain it to him.’ [P3]

A higher proportion of individuals in the PGD/PCBD group reported experiencing intrusive imagery relating to the moment of death (50.0%) compared with the NoPGD/PCBD group (18.7%).

Moment of death:

‘I see him lying in the hospital bed. When he was in hospital, he always wore a T-shirt. And then because he’d gone into heart failure the day before they put one of these horrible hospital gowns on him. And it’s white with black squares on it. And because it was so alien, he’d always been in his own clothes … And it sort of just brings it home that he’s not going to home. He’s not going to come back.’ [P25]

##### 3.2.1.2. Illness-related

Illness-related distressing intrusions were reported by 100.0% of the PGD/PCBD group and 81.2% of the NoPGD/PCBD group. An example within this theme related to the physical deterioration of the deceased due to illness and was described by 66.7% of the PGD/PCBD compared with only 25.0% of the NoPGD/PCBD group.

Physical deterioration:

‘I see him dribbling, he lost sort of the use of his lips and he use to dribble an awful lot, he always had to have a tissue and this snood would keep it there.’ [P7]

Other common examples of illness-related imagery included memories in which the deceased was in distress, and incidents of poor communication or lack of care from medical staff.

Deceased in distress:

‘He was in hospital for a draining around his lungs, and he’d got very stressed. It was one single thing that really broke him, he was watching the football on his phone and we’d let two goals in the first five minutes and I remember he’d normally be angry but this time he just started sobbing and saying I’m so stressed and threw his phone away.’[P2]

Poor communication/care of medical staff:

‘The medical people kept saying our aim is to get you back onto chemotherapy. This young doctor was only the same age as my son and he came from the same part of the country. And they got on… they were very friendly with each other, it was very nice. He whipped the curtain back. He says “I'm going off shift now but I just want to say to you if your heart stops, I don’t want to resuscitate you because you'll be a vegetable”#x2026A; And it frightened him, obviously. And I thought that was breathtakingly diabolical.’ [P13]

**Table:** 

Participant:	‘The medical people kept saying our aim is to get you back onto chemotherapy. This young doctor was only the same age as my son and he came from the same part of the country. And they got on … they were very friendly with each other, it was very nice. He whipped the curtain back. He says “I’m going off shift now but I just want to say to you if your heart stops, I don’t want to resuscitate you because you’ll be a vegetable” … And it frightened him, obviously. And I thought that was breathtakingly diabolical.’ [P13]

Two non-treatment-related intrusive imagery subordinate themes were extracted from the data – positive past memories and deceased in the present.

##### 3.2.1.3. Positive past memories

The majority of participants (87.5%) in the NoPGD/PCBD group reported spontaneous positive memories from the past compared to half of the PGD/PCBD group (50.0%). This participant describes an unexpected holiday she took with her wife.

‘It was her 30th. And it was a secret. And I took her to Florence. And we had no money. And we were supposed to be staying with friends. But when we got there, this posse of lesbians that we’ve never met have all arrived at the station and said, ‘we’re taking you to the countryside!’ And we were a bit like, but we’re suppose to be having our romantic holiday together. There was no arguing with them … And everyone piles into one of those little Fiat, little teenie weenie little things. And they took us and it was this derelict place, a converted church in the Tuscan countryside. And daddy made wine and there’s daddy’s wine. And lesbians kept arriving, and we sang under a tree, and it was just brilliant. That was not the holiday that we thought we were going to get, but it was just brilliant.’ [P24]

##### 3.2.1.4. Deceased in the present

Another subordinate theme of non-treatment related intrusions was defined by images of the deceased in the present (PGD/PCBD = 8.3%, NoPGD/PCBD = 25.0%). This participant describes intrusive imagery of her husband appearing wherever she goes.

‘He used to go to work in his black trousers and his Polo shirt. That was the uniform he had to wear and you know, I can still you know, I can just be sitting here and see it and I don’t even have to be sitting in my living room. It’s just so vivid.’ [P15]

#### 3.2.2. Qualities of memories

The superordinate theme qualities of memory refers to the characteristics or features of loss-related memories that seems to distinguish them from other memories. It contains six subordinate themes that describe particular aspects of loss-related memories i.e. memories relating to the death or the person that died. These themes were ‘negative memories take precedent’, ‘upsetting’, ‘happy memories cause pain’, ‘vividness’, ‘nowness’, and ‘felt presence’.

##### 3.2.2.1. Negative memories take precedent

The majority of the PGD/PCBD group (58.3%) and a proportion of the NoPGD/PCBD group (25%), reported that spontaneous pleasant memories were less frequently recalled than unpleasant memories. Those in the PGD/PCBD group described trying to pull positive memories into their consciousness to counteract the negative memories they were experiencing:

‘At the moment you know, what is absolutely full frontal in my brain is those last six months I spent with him. So it’s very, very difficult for me to go back beyond that to … I mean as I said, there’s one or two little holidays that I always try and recall because they’re the good times. So I do try and recall those memories as often as I can.’[P15]

Interviewer: ‘So they don’t arrive spontaneously?’

‘No. I have to actively seek that out. Because otherwise my memory of him are just in the awful, awful, awful last six months of his life and I don’t want it to be like that.’[P15]

Interviewer: ‘And you said at the beginning that it’s largely happy memories that come to your mind about your wife. But you’ve mentioned a few that actually sound very painful. How often do these memories come up at the moment?’

‘Well usually at the same time as I feel happy. I think of the bad things first and then I think of happy things. That gets me off it.’[P21]

Interviewer: ‘So you think of the last four weeks of her life first and then actively try and move on to something happier?’

‘Yes I go back to a few weeks before, I go back to when she was happy.’[P21]

**Table:** 

Participant 15:	‘At the moment you know, what is absolutely full frontal in my brain is those last six months I spent with him. So it’s very, very difficult for me to go back beyond that to … I mean as I said, there’s one or two little holidays that I always try and recall because they’re the good times. So I do try and recall those memories as often as I can.’
Interviewer:	‘So they don’t arrive spontaneously?’
Participant 15:	‘No. I have to actively seek that out. Because otherwise my memory of him are just in the awful, awful, awful last six months of his life and I don’t want it to be like that.’
Interviewer:	‘And you said at the beginning that it’s largely happy memories that come to your mind about your wife. But you’ve mentioned a few that actually sound very painful. How often do these memories come up at the moment?’
Participant 21:	‘Well usually at the same time as I feel happy. I think of the bad things first and then I think of happy things. That gets me off it.’
Interviewer:	‘So you think of the last four weeks of her life first and then actively try and move on to something happier?’
Participant 21:	‘Yes I go back to a few weeks before, I go back to when she was happy.’

##### 3.2.2.2. Upsetting

Two thirds (66.7%) of the PGD/PCBD group reported experiencing their loss-related memories as immensely, painful, and distressing compared with 44.8% of the NoPGD/PCBD group. Participants referred to loss-related memories as being both emotionally and physically distressing. This participant describes his reaction to a recurrent intrusive image of his mum in her hospital bed:

‘I feel it in my chest and in my body, I just feel sick. My heart pounds, and I get sharp pains. Yeah … this overwhelming feeling that the pit of your stomach kind of knots. It’s like you are hungry but you couldn’t eat. A sort of gnawing distress.’ [P28]

##### 3.2.2.3. Happy memories cause pain

A proportion of participants (25.0% of the PGD/PCBD group; 18.5% of the NoPGD/PCBD group) reported happy memories in the early stages of their bereavement being closely associated with loss and pain as they served to remind the individual of the deficit between the happiness in the past memory and the loss of the deceased the present moment.

‘Well people talk about ‘oh you’ve got your memories’ but they’re sad memories, they’re not happy memories now because he’s gone, so. They’ve now become very sad memories because it’s stopped, all the things we used to enjoy have stopped.’[P2]

##### 3.2.2.4. Vividness

Both groups similarly reported their loss-related memories as being vivid in nature (PGD/PCBD 41.7%; NoPGD/PCBD 43.8%).

‘Well it’s vivid, yes very vivid. I could almost describe the colours of the blanket, I think. She has a sort of, like skullcap on to keep her hear warm. It was green.’ [P9]

##### 3.2.2.5. Nowness

Although related to vividness, in that all memories with qualities of ‘nowness’ were reported as also vivid, a small proportion of participants reported a sense that the memory was happening again in the present moment or that it felt as though they were back reliving the memory (PGD/PCBD 8.3%; NoPGD/PCBD 18.8%).

‘It’s completely sensate. So it smells, I can smell that hospital, the old urine and the carpet underneath, the smell of cancer, the look of cancer, the greyness … I start to feel like I’m in the hospice.’ [P14]

##### 3.2.2.6. Felt presence

This theme is related to the intrusive imagery subordinate theme ‘deceased in the present’ but extends it by assessing whether these images were associated with a felt sense that the deceased was present in the here and now. A third of the PGD/PCBD (33.3%) and a quarter of the NoPGD/PCBD (25%) reported feeling as though the deceased were here when recalling images or memories of them.

‘It was like she was suddenly in the room, but as clear, clear as I can feel you. And she comes with different energies. Very playful in the beginning, really playful. Just a couple of times really tender – really, really tender holding. Couple of times I’ve seen her weep. And when I see her, it’s not like I see a ghost or a hologram. It’s … There’s the feeling of her energy, and then I get a visual image, but it’s like that’s put onto the energy.’ [P24]

#### 3.2.3. Triggers

Nine subordinate themes were used to describe triggers to loss-related memories and strong grief reactions: Time markers, couples and families, illness or death-related, internal physical and emotional triggers, music, possessions and photographs, shared situations, events or activities, ‘they would have done/thought/been here for that’, and when distraction comes to an end.

##### 3.2.3.1. Time markers

The subordinate theme time markers was reported by both the PGD/PCBD group (50.0%) and the NoPGD/PCBD group (31.3%) and references extracts to a particular time of day or year acting as a trigger to difficult memories. An example of the way that time can act as a trigger is seen in this participant’s descriptions of the impact of the calendar in which every day is an anniversary linked to previous years’ events during the deceased’s treatment:

‘And I just see every day as it was really. Tomorrow will be the day that they transported him back to his room, and then the grandchildren who came, who saw him, they just got out of the room and were crying in the corridor because they’d never seen him looking so unwell. I was there the whole time, you see, from morning until night, morning until night. And you know, it’s something which I can’t get out of my system. Probably if I get Alzheimer’s it will leave me.’ [P19]

##### 3.2.3.2. Couples and families

The majority of participants in the PGD/PCBD group (66.7%) reported being around or seeing couples and families as a trigger to memories and strong grief reactions in comparison to 37.5% of the NoPGD/PCBD group.

‘She invited everyone around for a big lunch and we all sat in the garden, and it was, I was the only single woman, I was the only single person. Everyone else was couples, of my age and [-]’s age and I found it really hard, I just, and I just … there were … times when I thought, how insensitive of you to invite me here and everyone’s sitting here talking about when they’re going to retire and what they’re going to do together as couples.’ [P26]

##### 3.2.3.3. Illness or death-related triggers

Excerpts from this theme describe situations, events, locations, activities, or objects that triggered specific illness or death related images. These included places like hospitals, journeys they took frequently when their loved one was sick or TV shows about death or cancer. Both groups reported this theme to a similar extent (PGD/PCBD 41.7%; NoPGD/PCBD 43.8%).

‘And then there’s also having a bath. Will always remind me of the time that she had fell unconscious after having a bath so you know I go to run the bath and I remember that. That’s almost every time I have a bath.’ [P20]

##### 3.2.3.4. Internal physical or emotional states

This theme relates to emotional states such as stress or vulnerability or internal physiological states such a tiredness or sickness acting as a trigger for loss-related memories or intense emotions and was reported by 50% of the PGD/PCBD group and 37.5% of the NoPGD/PCBD.

‘I suppose if someone’s being very mean at work or I’m with people I don’t like I guess I feel a little bit more lonely in the world. And then I realise that one of my greatest allies is gone and so I do connect those dots sometimes.’ [P27]

##### 3.2.3.5. Music

Music was reported as a trigger by both groups (PGD/PCBD 25.0%; NoPGD/PCBD 37.5%).

‘I listen to some music and I remember, you know, that perhaps … it brings back memories of what he might have said about it, or that he might have sung it or played it or whatever.’ [P19]

##### 3.2.3.6. Possessions or photographs

Possessions and photographs were reported as triggering deceased-related memories by over half of the PGD/PCBD group (58.3%) in comparison to 31.3% of the NoPGD/PCBD group.

‘But silly little things, like little teddy bears. I find those very comforting. An item of clothing, a bag. Again it’s the memories, they associate with memories. Even a little outfit she wore. I had a name for it because it was so funny, a little night-gown thing with shorts and things.’ [P6]

##### 3.2.3.7. Shared, locations, events, and activities

A majority of participants in both groups reported that locations, events and activities visited or attended together prompted memories. This theme is oriented in the past in that it generates memories of things that have happened (PGD/PCBD 58.3%; NoPGD/PCBD 62.5%).

‘Each time we go to the seaside, well, we’re going to Margate next, which [-] and I never went to, so he’s not there, but if we go back to Southwold, Norfolk, Cromer, he’s there. He’s all over the Gower.’ [P4]

##### 3.2.3.8. ‘They would have done/thought/been here.’

This theme contains exerts associated with current situations, events, or activities that the person who died would have liked, had an opinion on or would have taken part in/attended had they still been alive. It includes important events such as graduations and life successes and was reported by 58.3% of the PGD/PCBD group compared with 81.3% of the NoPGD/PCBD group.

‘I see film he might have enjoyed, and think oh, he should have been able to see that … I think triggers around things that he enjoyed doing and when the schools go back, like, in September the school went back and you, kind of, feel like, oh, he should be doing that. I think it definitely is a trigger.’[P5]

##### 3.2.3.9. When distraction comes to an end

This theme, reported similarly in both groups (PGD/PCBD 58.3%; NoPGD/PCBD 56.3%), describes a rise in their loss-related memories and strong emotions following periods of being engaged in something else such as sleeping, being at work, keeping busy with tasks, reading or watching television, and going out with others. Here a participant describes the experience of distraction coming to an end:

‘Even reading a book and enjoying reading it. Then you put it down and you’re back in an empty space. That you’ve taken your mind completely into some fictional thing, and maybe enjoyed following that piece of fiction, whether it’s a film or a play … you either go to the cinema on your own or you go together and then the lights go up and you’re back going out looking for your car or something. And suddenly it’s that lights up … reality. Reality was a nice place before, and it isn’t now. You know, reality was a good place, the lights up was a nice place to be because we were in a happy relationship and we were happy. Now, the lights up is saying, the whole world’s empty. It’s just you in it. And the longer the film or the book, the bigger the bounce at the other end.’ [P23]

## 4. Discussion

Semi-structured interviews with bereaved individuals with and without probable PGD/PCBD (American Psychiatric Association (Producer), 2012; Prigerson et al., 2009) who had lost a person important to them to cancer resulted in three superordinate themes in response to questions about loss-related memories (i.e. intrusive imagery, qualities of memory, and triggers).

The superordinate theme of intrusive imagery contained four subordinate themes of memory content (change for the worse/loss of hope, illness-related imagery, positive past memories, deceased in the present). These themes described images that, at the time of the interview, participants were experiencing intrusively and repeatedly. While intrusive images have been investigated previously during bereavement (Boelen & Huntjens, 2008) few studies have reported intrusive memories following cancer loss (Sanderson et al., 2013) and none have reported differences between those with and without PGD/PCBD. Almost everyone in the sample, irrespective of group, reported experiencing intrusive loss-related imagery. The PGD/PCBD group reported more treatment-related imagery (e.g. change for the worse/loss of hope, illness-related imagery) and less spontaneous positive past imagery than the NoPGD/PCBD group. When describing qualities of memory, they also more frequently reported involuntary memories to be predominantly negative in nature. This may suggest people with PGD/PCBD demonstrate bias towards loss-related memories (Maccallum & Bryant, 2010a) which likely interferes with the spontaneous activation of positive memories of the deceased. Those in the PGD/PCBD group more readily reported having to deliberately recall positive memories, a process that they described as effortful. The lack of spontaneous positive reminiscing is likely to contribute to feelings of low mood and a lack of psychological closeness to the deceased that in turn has the potential to trigger intense pain and grief. Given the differences between the PGD/PCBD and the no-PGD/PCBD groups, it is possible that as grief adapts over time, and the loss becomes integrated with other autobiographical memories, negative intrusive imagery naturally resolves allowing for more positive reminiscing. Other subordinate themes describe loss-related memories as vivid, upsetting, and associated with a felt sense that the deceased is present. Although less common some participants also reported memories to have a ‘nowness’ qualities that led them to feel as though the event was happening again or that they were back experiencing the event again. Memories that are vivid, distressing, and have a sense of ‘nowness’ interestingly parallels the characteristics of memory described by people with posttraumatic stress disorder (PTSD) (Hackmann, Ehlers, Speckens, & Clark, 2004; Michael, Ehlers, Halligan, & Clark, 2005). A psychological disorder experienced after a traumatic event, which can include, but is not exclusive to, bereavement.

The final superordinate theme described a variety of triggers of loss-related memories or strong grief reactions. The largest discrepancy between groups was found in the couples and families theme with the PGD/PCBD group reporting seeing or spending time with couples and families as triggering more frequently than the NoPGD/PCBD group. Excerpts in this theme often contained the sentiment that participants were aware of coming into contact with couples and families more often than they had previously. Previous research in PTSD has suggested that sufferers of PTSD more readily identify trauma-related stimuli in their environment than those without PTSD and that this enhanced priming effect is associated with symptom severity (Michael, Ehlers, & Halligan, 2005), therefore it is possible that a similar process is relevant in PGD/PCBD. Another notable difference was the frequency of ‘they would have done/thought/been here’ trigger with the vast majority of participants in the NoPGD/PCBD group reporting this theme compared with just over half of the PGD/PCBD group. It may be that being able to imagine the deceased’s opinion on things happening in the present moment represents an enduring psychological connection to the deceased without their physical presence. Worden described the task of remaining connected to the deceased as a goal of grief work (Worden, 2010), and it may be that this process is easier for those who do not suffer from PGD/PCBD.

In terms of demographic and loss characteristics, only level of education differed between groups. This is in line with a recent meta-analysis that found higher educational level to be associated with lower PGD severity (Heeke, Kampisiou, Niemeyer, & Knaevelsrud, 2017).

These findings add to our understanding of the content, triggers and qualities of loss-related memories following cancer bereavement and how loss-related memories are related to PGD/PCBD. However, there are some limitations that should be considered. The investigator, a clinical psychologist, used the interview schedule to guide areas of questioning but would explore antecedents, behaviours, and consequences of a particular experience to gain insight into factors relevant to a CBT model. As such concepts that may be relevant to grief memories from a different theoretical framework, such as childhood memories, were not explicitly explored. Future research focusing on the impact of childhood experiences on adult grief reactions would help bridge the gap between the line of questioning reported here and formative attachment relationships. As no validated measures of PCBD exist, we had to represent the criteria with items from the PG-13 and additional items. Owing to the small sample size, codes were not subject to significance testing. Future research would benefit from a larger sample to determine whether these groups differ significantly in terms of loss-related memories.

Finally, this sample consisted solely of participants bereaved by expected natural causes. Individuals bereaved by sudden deaths often do not have the time to ready a support network nor are they already in contact with services able to provide support such as a hospice or palliative care setting (Rodger, Sherwood, O’Connor, & Leslie, 2007). This delay may mean that sudden deaths prove higher risk with respect to long-term health consequences for the bereaved. Future research comparing the experiences of natural and sudden deaths would likely shed light on whether the content and characteristics of loss-related memories differs by mode of death.

This is the first qualitative study to compare the features of loss-related memories in a sample of those with and without probable PGD/PCBD. Our results suggest that both groups report negative intrusive imagery, however it was more common in the PGD/PCBD group. Clinical approaches that include memory processing (Bryant et al., 2014; Ehlers & Clark, 2000; Resick, Monson, & Chard, 2016) may be of particular relevance to those bereaved by cancer.

## Supplementary Material

Supplemental Material

## Note

1. For details of PCBD items please see supplementary material.

2. See supplementary material.
