# Supplementary material for: A qualitative analysis of loss-related memories after cancer loss: a comparison of bereaved people with and without prolonged grief disorder
Source: Eur J Psychotraumatol. 2020 Sep 23;11(1):1789325. doi: 10.1080/20008198.2020.1789325 (PMC7534291; doi:10.1080/20008198.2020.1789325)
Supplement: Supplemental Material [file ZEPT_A_1789325_SM6797.pdf]

PCBD conceptualisation

Two items were added to the PG-13 scale to assess criterion B of PCBD 'how often have you been preoccupied with thoughts or memories of the deceased?' and 'how often have you been preoccupied with thoughts about the circumstances of the death?' Four items were added to criterion C 'do have difficult recalling happy memories that involve the deceased?', 'do you feel bad about yourself because of things that happened in relation to the death or between you and the deceased?', 'do you feel that a desire to die to be with the deceased?', 'do you feel alone or detached from others since the death?' The PCBD symptom 'feeling shocked, stunned or emotionally numb' was considered present if PGD item 5 (How often have you felt stunned, shocked or dazed by your loss?) or PGD item 11 (Do you feel emotionally numb since your loss?) were reported as present.
